# Numerical Study of Ignition and Combustion of Hydrogen-Enriched Methane in a Sequential Combustor

**DOI:** 10.1007/s10494-024-00540-8

**Published:** 2024-04-05

**Authors:** Matteo Impagnatiello, Quentin Malé, Nicolas Noiray

**Affiliations:** CAPS Laboratory, Department of Mechanical and Process Engineering, ETH Zürich, 8092 Zurich, Switzerland

**Keywords:** Sequential combustor, Hydrogen blending, Turbulent combustion, Gas turbine for power generation, Combustion regime

## Abstract

Ignition and combustion behavior in the second stage of a sequential combustor are investigated numerically at atmospheric pressure for pure ${\text{CH}}_{4}$ fueling and for two ${\text{CH}}_{4}$-${\text{H}}_{2}$ fuel blends in 24:1 and 49:1 mass ratios , respectively, using Large Eddy Simulation (LES). Pure ${\text{CH}}_{4}$ fueling results in a turbulent propagating flame anchored by the hot gas recirculation zones developed near the inlet of the sequential combustion chamber. As the ${\text{H}}_{2}$ content increases, the combustion process changes drastically, with multiple auto-ignition kernels produced upstream of the main flame brush. Analysis of the explosive modes indicates that, for the highest ${\text{H}}_{2}$ amount investigated, flame stabilization in the combustion chamber is strongly supported by auto-ignition chemistry. The analysis of fuel decomposition pathways highlights that radicals advected from the first stage flame, in particular OH, induce a rapid fuel decomposition and cause the reactivity enhancement that leads to auto-ignition upstream of the sequential flame. This behavior is promoted by the relatively large mass fraction of OH radicals found in the flow reaching the second stage, which is approximately one order of magnitude greater than it would be at chemical equilibrium. The importance of the out-of-equilibrium vitiated air on the ignition behavior is proven via an additional LES that features weak auto-ignition kernel formation when equilibrium is artificially imposed. It is therefore concluded that parameters affecting the relaxation towards chemical equilibrium of the vitiated flow can have an important influence on the operability of sequential combustors fueled with varying fractions of ${\text{H}}_{2}$ blending.

**Supplementary Information:**

The online version contains supplementary material available at 10.1007/s10494-024-00540-8.

## Introduction

A new type of combustor architecture for heavy duty gas turbines has emerged recently: the Constant Pressure Sequential Combustor (CPSC) (Pennell et al. 2017). This technology is a step forward in the design of operationally- and fuel-flexible gas turbines (Ciani et al. 2019; Güthe et al. 2008) that can cope with both the intrinsically intermittent nature of renewable energy production and with the increasing demand in the power generation sector to burn different fuel blends, and in particular blends incorporating a large amount of sustainably produced hydrogen. The CPSC concept consists of two technically premixed flames burning in series. No turbine row is positioned between the two stages, differently from previous re-heat designs, making the pressure across the sequential combustor quasi-uniform. In this architecture, hot flue gases provided by the lean first stage flame are diluted with compressor air upstream of the sequential stage. The role of the dilution air is twofold: it increases the O_2_ content to completely burn the gas in the sequential stage, and it decreases the gas temperature, thus ensuring sufficiently long autoignition time for good mixing in the sequential burner and therefore very low NO_x_ emissions.

The two flames burn at very different thermodynamic conditions, resulting in different combustion modes. The first stage exhibits a purely propagating turbulent flame which is aerodynamically anchored in the combustion chamber as in conventional single stage gas turbine combustors. The reactants are characterized by a relatively low temperature, far below the auto-ignition temperature. In contrast, the temperature of the reactants in the sequential stage is significantly higher, allowing auto-ignition chemistry to play a role in the combustion process (Schulz and Noiray 2018; Ebi et al. 2019; Aditya et al. 2019; Gruber et al. 2021; Savard et al. 2019).

Auto-ignition chemistry can significantly affect different aspects of the combustion process, such as mixture reactivity and flame speed (Savard et al. 2020), with consequences on the acoustic response of the combustor (Schulz and Noiray 2018). The CPSC architecture was designed for high fuel flexibility and enables low NO_x_ combustion of ${\text{CH}}_4$–${\text{H}}_2$ blends with large mass fractions of ${\text{H}}_2$ (Ciani et al. 2019). However, for off-design locations, the flame may undergo an early ignition in the sequential burner mixing section, potentially increasing NO_x_ due to mixture stratification, and damaging the burner if the flame anchors at locations that are not designed to sustain high thermal loads (Schmalhofer et al. 2018).

This peculiar behavior sets different challenges in the design of such combustors operating at auto-ignition conditions. An in-depth understanding of the relevant combustion physics is therefore required. Multiple studies have contributed to the problem of auto-ignition in hot turbulent environments in different configurations and with different fuels (e.g., Diamantis et al. 2015; Sidey et al. 2014; Sidey and Mastorakos 2015; Deng et al. 2016; Novoselov et al. 2021). The combustion behavior of hydrogen at auto-igniting conditions in a sequential combustor configuration has been experimentally investigated in our laboratory in Solana-Pérez et al. (2022a), Solana-Pérez et al. (2022b). The present study builds on this latter work, but focuses on the effects that hydrogen enrichment has on methane combustion.

The addition of hydrogen to natural gas is promising for sequential combustor architectures, as they were found capable of handling extreme variations of ${\text{H}}_{2}$ content at gas turbine baseload conditions with minor downgrades in combustor performances (Bothien et al. 2019; Ciani et al. 2020, 2021). In this respect, there is a need to develop predictive tools for the effect of fuel composition variations, which affect auto-ignition delays, flame dynamics, and pollutants formation, motivating the research efforts on the topic (e.g., Fleck et al. 2012; Berger et al. 2018).

In this regard, the objective of the present work is to investigate the effects and the challenges of ${\text{H}}_{2}$ blending on the combustion process in the second stage of a sequential combustor using numerical tools. Large Eddy Simulation (LES) associated with Analytically Reduced Chemistry (ARC) for the description of the chemical kinetics is used to perform the computations. Analysis of the explosive modes introduced by chemical kinetics is used to characterize the combustion regimes. The influence of radicals advected from the first stage flame on the auto-ignition chemistry is examined via Reaction Path Analysis (RPA), and the importance of the relaxation towards chemical equilibrium of the vitiated air flow is investigated via an additional ad-hoc LES.

## Computational Setup

### Computational Domain and Operating Points

The configuration investigated in this work is a generic sequential combustor used for research at ETH Zürich. Since the physical phenomena of interest occur in the second stage, only this part of the combustor is simulated, at atmospheric pressure.

**Fig. 1:**
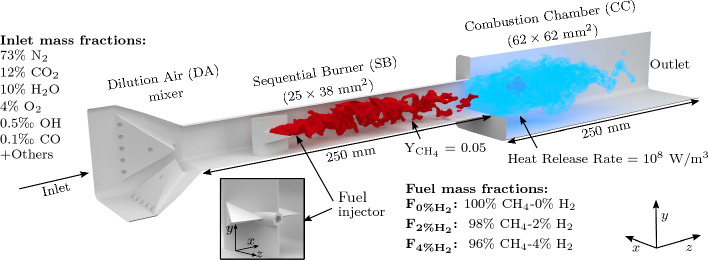
Diagram of the second stage of the sequential combustor used for the three-dimensional LES

The simulated sequential stage is shown in Fig. 1 and consists of three parts: the Dilution Air mixer (DA), the Sequential Burner (SB) and the Combustion Chamber (CC). The DA module connects the first stage combustion chamber with the SB. It is equipped with large lateral vortex generators and multiple air injection holes for rapid mixing. The axial fuel injector is located inside the SB. Its shape is an extruded, symmetric airfoil with two vortex generators attached on each side to favor mixing between the fuel and the co-flow of hot vitiated air.

In the present configuration, the inlet mixture consists of 15.7 g/s of combustion products from a lean premixed methane-air flame burning at an equivalence ratio of 0.8 in the first stage, resulting in the composition outlined in Fig. 1. The imposed inlet velocity profile follows a power law with coefficient 7. The inlet temperature is set to 1860 K, which corresponds to 93% of the adiabatic flame temperature, in order to take into account the heat losses at the walls of the first stage combustion chamber. The amplitude of this temperature drop is estimated on the basis of previous experimental results (Weilenmann et al. 2021), where quantitative OH-LIF thermometry was used downstream of two different technically-premixed first stages: a $4\times 4$ array of jet flames and a swirled flame. Chemical equilibrium at 1860 K is assumed to set the gas composition at the inlet of the simulated sequential stage.

The inlet mixture is diluted in the DA mixer with 8.5 g/s of air at ambient temperature, leading to a globally lean combustion in the sequential stage. The fuel mass flow in the sequential stage is 0.6 g/s. Throughout this study, all the parameters discussed above are fixed. Three cases are simulated, which differ only in terms of sequential fuel composition:

- case ${\text{F}}_{{0\%{{\rm H}}_{2}}}$ where the fuel is pure ${\text{CH}}_{4}$, resulting in an equivalence ratio of 0.9;
- case ${\text{F}}_{{2\%{{\rm H}}_{2}}}$ where the fuel is a CH_4_–H_2_ blend with 2% ${\text{H}}_{2}$ in mass (14% in volume), resulting in an equivalence ratio of 0.92 based on both ${\text{CH}}_{4}$ and ${\text{H}}_{2}$;
- case ${\text{F}}_{{4\%{{\rm H}}_{2}}}$ where the fuel is a CH_4_–H_2_ blend with 4% ${\text{H}}_{2}$ in mass (25% in volume), resulting in an equivalence ratio of 0.94 based on both ${\text{CH}}_{4}$ and ${\text{H}}_{2}$.

The hydrogen addition results in an increase in the flame thermal power of 2.8 and 5.6% for ${\text{F}}_{{2\%{{\rm H}}_{2}}}$ and ${\text{F}}_{{4\%{{\rm H}}_{2}}}$, respectively. The walls of the domain are defined as isothermal. Two types of walls exist in the experiments: air-cooled quartz walls and water-cooled aluminium walls. In the simulations the surface temperature is set to 1000 K for the quartz walls and 700 K for the aluminium walls. The latter walls are located in the DA mixer and at the junctions between the different elements; the former walls are located in the SB and in the CC. The fuel injector is also made of aluminium but is heavily water-cooled. Therefore, its surface temperature is set to 400 K in the simulations. These temperatures are estimated from conjugate heat transfer simulations based on steady Reynolds Averaged Navier–Stokes (RANS) solutions at nominal operating conditions. These simulations were performed during the conception of the experimental sequential combustor using the commercial code “Solidworks Flow Simulation”, and they incorporate both water and air cooling systems. The applied model involves a homogeneous, non-reacting flow, with imposed temperature matching the expected conditions during combustor operation.

### Large Eddy Simulation Setup

LES are performed using the explicit massively parallel compressible solver AVBP (Schönfeld and Rudgyard 1999; Gicquel et al. 2011). The numerical framework used in this work has been experimentally validated in Schulz et al. (2019a), Malé et al. (2022), Malé et al. (2024) for similar sequential combustor geometries. Moreover, this work shows that the results remain unchanged after a mesh refinement for the chosen grid size. The present LES results are therefore considered a trustworthy database for the type of analysis and the purpose of this work.

A fully explicit two-step Taylor–Galerkin finite element numerical scheme (Colin and Rudgyard 2000) is used, which offers third order accuracy in space and time. The sub-grid scale turbulence effect is modeled using the SIGMA model (Nicoud et al. 2011). Classical law-of-the-wall modeling is used to account for the unresolved boundary layer. The following formulation for the non-dimensional velocity $u^+$ is employed:

1 $$\begin{aligned} {\left\{ \begin{array}{ll} u^+={u} / {u_\tau }=y^+ &{} {\text{for}}\; y^+ \le 11,\\ u^+={u} / {u_\tau }=\frac{1}{\kappa } \ln {(E y^+)} &{} {\text{for}}\; y^+ > 11 \text {,}\\ \end{array}\right. } \end{aligned}$$

where *u* is the flow velocity, ${u_\tau }$ is the friction velocity $u_{\tau }=\sqrt{{\tau _w} / {\rho }}$ with $\tau _w$ being the wall shear stress and $\rho$ being the gas density, $y^+$ is the non-dimensional wall distance $y^+={y \, u_\tau } / {\nu }$ with *y* being the distance to the wall and $\nu$ being the fluid kinematic viscosity, $\kappa =0.41$ and $E=9.2$. The Navier–Stokes Characteristic Boundary Conditions (NSCBC) formulation (Poinsot and Lele 1992) is applied to the inlets and the outlet of the domain. The Dynamic Thickened Flame (DTF) model is used to model turbulent combustion (Legier et al. 2000). Unresolved flame wrinkling effects are modeled using the efficiency function defined in Charlette et al. (2002). To ensure that the auto-ignition is not biased by the DTF model in the sequential burner (Schulz et al. 2017; Schulz and Noiray 2019b), the activation of the model is limited to the combustion chamber only as done in Malé et al. (2022), Malé et al. (2024). An applied Thickening Factor (TF) of 7 ensures that $\approx 5$ grid points are located inside the flame front.

The chemical kinetics relies on Artificially Reduced Chemistry (ARC). The ARC mechanism consists of 19 transported species, 8 species in quasi-steady state approximation and 139 reactions. This mechanism has been reduced from the GRI3.0 mechanism (Smith et al. 1999) using the ARCANE reduction tool (Cazéres et al. 2021), and has been validated via zero-dimensional (0D) and one-dimensional (1D) Cantera (Goodwin et al. 2022) simulations.

The computational mesh consists exclusively of tetrahedral elements and was generated using Gmsh (Geuzaine and Remacle 2009).

The simulation data are collected over 10 ms, a duration that was found adequate to achieve converged statistics. The resulting computational cost per simulated case is of around $100 \, 000$ CPU-hours, excluding the transient period to reach a statistically steady state.

**Fig. 2:**
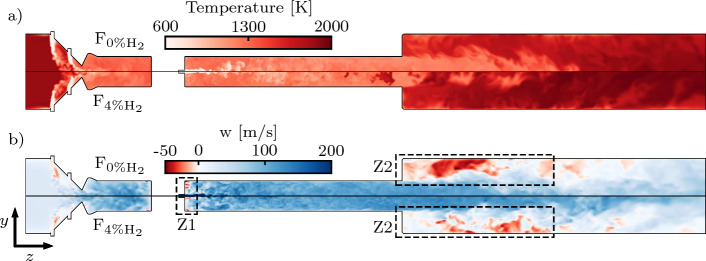
Planar cut colored by the temperature (**a**) and the streamwise velocity *w* (**b**). The top halves represent the F_0%H2_ case; the bottom halves represent the F_4%H2_ case. The recirculation zones Z1 and Z2 are highlighted by the dashed rectangles

Figure 2 provides the instantaneous fields of temperature and axial velocity obtained from LES under F_0%H2_ and F_4%H2_ conditions. The intermediate F_2%H2_ results are omitted for brevity. The DA mixer allows a rapid temperature decrease before the sequential fuel injector. The presence of hot temperature spots at this location is thereby minimized. Despite the high turbulence intensity produced by the vortex generators, temperature and composition stratification persist in the SB for the present configuration. Fuel mixing with the co-flow occurs through the whole length of the SB. The streamwise velocity field highlights the presence of two critical recirculation zones. The first one (Z1), located right after the fuel injector, supports the formation of a chemically-active region that promotes fuel decomposition and that will be scrutinized in Sect. 3.2. The second recirculation zone (Z2) is located in the CC and strengthens the flame stabilization. Hot combustion products and radicals are trapped in this recirculation zone and support flame anchoring at the burner outlet, thereby inhibiting sudden flame blow-off when the ignition delay becomes significantly longer than the residence time in the SB (Ebi et al. 2019; Schulz and Noiray 2019a).

### Chemical Explosive Mode Analysis

Analysis of the explosive modes introduced by chemical kinetics is performed using Chemical Explosive Mode Analysis (CEMA) algorithmic tools (Lu et al. 2010). As meticulously described in Goussis et al. (2021), the algorithmic tools incorporated into CEMA were developed on the basis of the ones defined within the Computational Singular Perturbation (CSP) framework (Lam and Goussis 1989), that has been employed for the development of stiff ODE solvers (Valorani and Goussis 2001; Debusschere et al. 2012), reduction of chemical mechanisms (Massias et al. 1999; Lee et al. 2007; Valorani et al. 2006) and physical understanding of combustion process (Valorani et al. 2003; Prager et al. 2011). CEMA is based on the eigenanalysis of the Jacobian of the local chemical source term $\mathbf {J_{\omega }}$ in the governing equation of a reacting flow

2 $$\begin{aligned} \frac{ D \varvec{\omega } ({\varvec{y}}) }{ D t } = \mathbf { J_{\omega } } \frac{ D {\varvec{y}} }{D t} = \mathbf { J_{\omega } } (\varvec{\omega } + {\varvec{s}}) \, \text {,} \quad \mathbf {J_{\omega }} = \frac{\partial \varvec{\omega }}{\partial {\varvec{y}}} \, \text {,} \end{aligned}$$

where ${\varvec{y}}$ is the vector of local thermochemical state variables including temperature and species concentrations, $\varvec{\omega }$ is the vector of the local chemical source terms, and ${\varvec{s}}$ is the vector of the local non-chemical source terms (e.g., diffusion). Being interested in the processes leading to ignition, the mode analysis is restricted to the explosive modes, i.e. the ones whose associated eigenvalue has a positive real part and which will tend to drive the system away from equilibrium in an isolated environment. In contrast to CSP, CEMA assumes that the dynamics of the reactive mixture is unconditionally controlled only by the fastest explosive mode, i.e., the Chemical Explosive Mode (CEM), for which

3 $$\begin{aligned} \Re \left( \lambda _e\right) >0, \quad {\mathbf {J_{\omega }}\varvec{b_e}=\lambda _e\varvec{b_e}, \quad \varvec{a_e}\mathbf {J_{\omega }}=\lambda _e\varvec{a_e},} \quad \Re \left( \lambda _e\right) =\max _{i}\left( \Re \left( \lambda _i\right) \right) \, \text {,} \end{aligned}$$

where $\lambda _e$, $\varvec{a_e}$, and $\varvec{b_e}$ indicate the eigenvalue, the right eigenvector, and the left eigenvector of the CEM, respectively, and $\lambda _i$ represents the eigenvalue associated with the $i^{\textrm{th}}$ mode. Given that the thermochemical state evolution is non-linear, the assumption of CEM dominance may not always hold. Particular attention will be paid to this subject when applying CEMA in the present work to ensure the relevance of the analysis. Furthermore, the existence of an explosive mode is purely a chemical property of the system. Non-chemical processes may still play a role in the evolution of the system, making the existence of a CEM a necessary but not sufficient condition for the investigated mixture to ignite/explode.

The Explosive Index ($\textbf{EI}$) vector, which has the same dimensions as the state vector ${\textbf{y}}$, can be defined as (Lu et al. 2010)

4 $$\begin{aligned} \textbf{EI} = \frac{|{\text{diag}}(\varvec{a_e}\varvec{b_e})|}{{\text{sum}}(|{\text{diag}}(\varvec{a_e}\varvec{b_e})|)} \, \text {.} \end{aligned}$$

$\textbf{EI}$ is a scaled CSP pointer, introduced in Goussis and Lam (1992). Each entry of the $\textbf{EI}$ vector, bounded between zero and unity, quantifies the contribution of the corresponding thermochemical property (either temperature or a species mass fraction) to the CEM. The $\textbf{EI}$ vector is analyzed in Sect. 3 to determine which thermochemical property has locally the largest effect on the CEM.

Projecting Eq. (2) in the direction of the CEM left eigenvector ${\varvec{b}}_e$, the following relation can be obtained (Xu et al. 2019a)

5 $$\begin{aligned} \frac{D \phi _{\omega }}{D t} = \lambda _e \phi _{\omega } + \lambda _e \phi _s + \frac{D \varvec{b_e}}{D t} \cdot \varvec{\omega }({\varvec{y}}) \end{aligned}$$

where the amplitudes of the projected chemical ($\phi _{\omega }$) and diffusion ($\phi _s$) source terms are defined as

6 $$\begin{aligned} \phi _{\omega } \equiv {\varvec{b}}_e \cdot \varvec{\omega } \, \text {,} \end{aligned}$$

7 $$\begin{aligned} \phi _s \equiv {\varvec{b}}_e \cdot {\varvec{s}} \, \text {.} \end{aligned}$$

The last term in Eq. (5) represents a nonlinear effect induced by the rotation of the eigenvector and is neglected in the present work.

A local combustion mode indicator ${\alpha }$ is then defined as (Xu et al. 2019a):

8 $$\begin{aligned} \alpha = \frac{\phi _s}{\phi _\omega } \, \text {,} \end{aligned}$$

for explosive mixtures (i.e., at least one eigenvalue has a positive real part). This ratio compares the relative alignments of the diffusion and chemical source terms with the CEM left eigenvector. It indicates the relative importance of chemistry and diffusion for the explosive mixtures, whose dynamics is assumed to be governed by the CEM. The value of ${\alpha }$ will be used in this work to describe the nature of the ongoing combustion process and reveal several combustion regimes:

(i) $\alpha >1$, assisted-ignition regime: diffusion dominates chemistry and promotes ignition;
(ii) $\alpha <-1$, extinction regime: diffusion dominates chemistry but acts against ignition;
(iii) $|\alpha |\le 1$, auto-ignition regime: chemistry plays a dominant role in ignition.

This indicator has been successfully used to distinguish between two propagation modes of 1D premixed flames: deflagration waves and auto-igniting waves (Xu et al. 2019a). The transport budget analysis that was carried out demonstrated the reliability of the indicator. Additionally, it has been successfully applied to the study of combustion regimes in many different configurations, e.g., strongly turbulent premixed flames (Xu et al. 2019b), jet in crossflow (Schulz et al. 2019b), reheat combustion (Aditya et al. 2019), moderate or intense low oxygen dilution combustion (Doan et al. 2021), deflagration to detonation transition (Jaravel et al. 2021), hot jet ignition (Malé et al. 2021), plasma-induced ignition (Malé et al. 2022).

In the present study, CEMA is employed as a post-processing step on the 3D instantaneous fields obtained from the LES, resulting in a computational cost of around 12 CPU-hours per instant.

### Validation of the Numerical Setup

#### ARC Mechanism

**Fig. 3:**
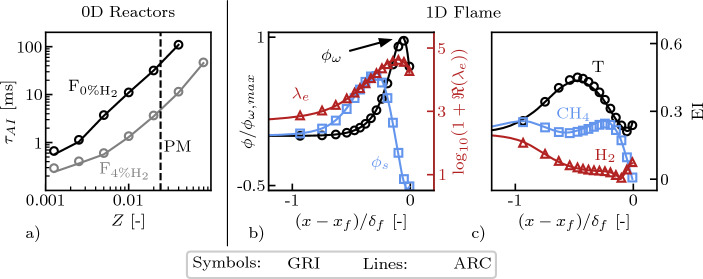
Comparison of the detailed (GRI) and the ARC mechanisms in terms of: auto-ignition time $\tau _{AI}$ for F_0%H2_ and F_4%H2_ conditions (**a**) and of important quantities related to the analysis of the explosive modes detailed in Sect. 2.3 for F_0%H2_ conditions (**b** and **c**). Only three entries of the $\textbf{EI}$ vector corresponding to temperature, ${\text{CH}}_{4}$, and ${\text{H}}_{2}$ are represented for brevity. The vertical, dashed line in (**a**) represents the Perfectly Mixed (PM) mixture fraction. $x_f$ is the flame location and $\delta _f$ the flame thickness

A great agreement in terms of both ignition delay and laminar flame speed is found between the detailed and the reduced mechanism. Ignition delays as function of the mixture fraction *Z* between the vitiated air and the second stage fuel are shown in Fig. 3a for the F_0%H2_ and F_4%H2_ fuel compositions. The thermodynamic properties of the vitiated air have been set taking the average temperature and mixture composition from a plane right upstream of the fuel injector in the LES. The reduced and detailed mechanism are also in agreement regarding the quantities related to the analysis of the explosive modes detailed in Sect. 2.3. The results for the real part of the explosive mode eigenvalue $\Re \left( \lambda _e\right)$ (Eq. (3)), for the amplitudes of the projected chemical $\phi _{\omega }$ (Eq. (6)) and diffusion $\phi _s$ (Eq. (7)) source terms, and for the components of the **EI** vector corresponding to temperature, CH_4_ and H_2_ (Eq. (4)) are shown in Figs 3b and c using a 1D laminar flame at F_0%H2_ conditions. The thermochemical state of the fresh gas is set assuming perfect mixing between the vitiated air and the fuel. The two mechanisms are in good agreement with each other also for other mixture fractions *Z* around the perfectly mixed one.

#### Grid Sensitivity Analysis

**Fig. 4:**
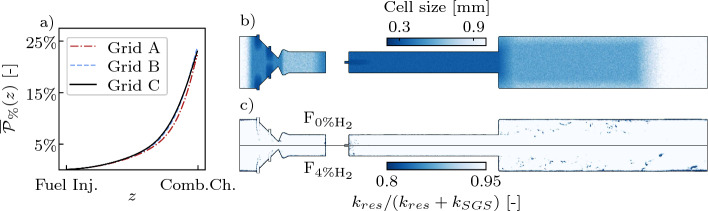
$\overline{{\mathcal {P}}}_{\%}$ (Eq. (9)) as function of the streamwise coordinate *z* in the SB at F_4%H2_ operating conditions for the three different grids (Table 1) used in the GSA (**a**). Planar cuts colored by the cell size (**b**) and by the ratio between the resolved $k_{res}$ and the total kinetic energy $k_{res}+k_{SGS}$ (**c**) for Grid B. The kinetic energy ratio is represented for two instantaneous solutions at F_0%H2_ (top half) and F_4%H2_ (bottom half) operating conditions

A Grid Sensitivity Analysis (GSA) has been performed to assess the capability of the LES grid to correctly capture the auto-ignition kernels formation and evolution in the SB, and to allow an accurate use of the diagnostic tools employed. Three different grids featuring progressively smaller grid characteristic cell sizes $\Delta _x$ in the SB have been generated for the GSA (Table 1). The metric used to evaluate the grids with respect to the auto-ignition kernel dynamics is the time-averaged thermal power released up to the streamwise coordinate *z* defined as

9 $$\begin{aligned} \overline{{\mathcal {P}}}_{\%}(z) = \frac{1}{t^{av} P_{2nd}^{th.}} \int _0^{t^{av}} \int _{-\infty }^z \iint {\dot{Q}}(x,y,z^*,t) \, dx \, dy \, dz^* \, dt \, \text {,} \end{aligned}$$

where ${\dot{Q}}$ is the heat release rate, $P_{2nd}^{th.}$ is the total power of the second stage, and $t^{av}=10$ ms is the averaging time. The evolution of $\overline{{\mathcal {P}}}_{\%}(z)$ is shown in Fig. 4 for the three different grids and the F_4%H2_ case. The grid no longer affects the metric when $\Delta _x \le 0.3$ mm. For F_0%H2_, no chemical activity is observed in the SB for any grid. Grid B can therefore be considered reliable to conduct this study. The independence of the results to a refinement is also assessed in terms of species mass fraction profiles and quantities related to the analysis of the explosive modes detailed in Sect. 2.3, and to the Reaction Path Analysis (RPA) performed in Sect. 3.2. This information is added as Supplementary Material.

**Table 1:** Characteristic cell sizes $\Delta _x$ and total number of cells of the grids used for the grid sensitivity analysis

Grid	$\Delta _x$ (µm)			Cells no. (mio.)
Name	DA	SB	CC	
Grid A	400	400	500	44
Grid B	400	300	500	62
Grid C	400	250	500	84

In addition, the quality of Grid B is assessed a-posteriori by computing the ratio between the resolved kinetic energy $k_{res}$ and the total kinetic energy, using a similar approach as in Schulz and Noiray (2018), Kempf et al. (2006). The sub-grid scale kinetic energy is estimated as (Deardorff 1980)

10 $$\begin{aligned} k_{SGS}={\nu _T}^2/(C_V \Delta )^2 \text {,} \end{aligned}$$

where the turbulent viscosity $\nu _T$ is extracted from the LES, $C_V=0.1$ (Pope 2000) is a constant, and the filter size $\Delta$ is estimated as the cubic root of the volume of each cell. The instantaneous values of the kinetic energy ratio $k_{res} / (k_{res}+k_{SGS})$ for the F_0%H2_ and F_4%H2_ simulations are shown in Fig. 4b, together with the mesh cell size. Numerical dissipation is not explicitly included in this ratio. Nevertheless, this simplification is expected to have limited effects due to the low-dissipative, high-order numerical scheme employed in this study. The kinetic energy ratio is greater than 80% almost everywhere in the domain, suggesting a good resolution of the turbulent spectrum within the LES framework (Pope 2000). $y^+$ values range approximately between 5 and 30, thus remaining within the validity range of the law-of-the-wall (Eq. (1)): no further refinement near the walls is needed. Grid B is ultimately chosen to conduct the study.

#### CEMA Combustion Mode Indicator

**Fig. 5:**
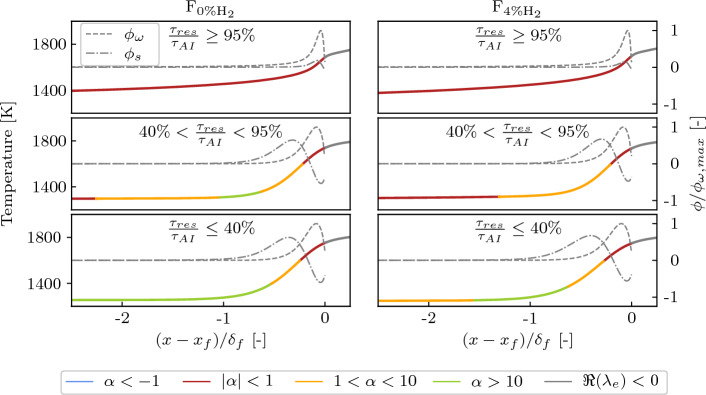
Profiles of temperature and projected chemical $\phi _\omega$ (Eq. (6)) and diffusion $\phi _s$ (Eq. (7)) source terms obtained from 1D premixed flames for different inlet residence times $\tau _{res}$. The temperature profile is colored by the value of the combustion mode indicator $\alpha$ (Eq. (8)), which depicts the change in combustion regime. The non-explosive mixture is indicated by $\Re (\lambda _e)<0$. Inlet thermochemical conditions are representative of F_0%H2_ (left) and F_4%H2_ (right). $x_f$ is the flame location and $\delta _f$ the flame thickness

The capability of the local combustion mode indicator $\alpha$ to distinguish between deflagration waves controlled by back-diffusion of heat and radicals and auto-ignition controlled reaction fronts is now assessed for the thermochemical conditions of the present work using 1D premixed flames computed using Cantera (Goodwin et al. 2022). The inlet thermochemical conditions of the 1D flames are set assuming perfect mixing between the vitiated air and the fuel (i.e., vertical line in Fig. 3a) at the F_0%H2_ and F_4%H2_ operating points. The results from the intermediate F_2%H2_ conditions are omitted for brevity. The combustion regimes are imposed by varying the ratio of the residence time between the inlet and the flame front ($\tau _{res}$) to the auto-ignition time ($\tau _{AI}$) of the unburned mixture. In practice, this is accomplished by modifying the length $l_D$ of the 1D domain. Since the Cantera solver always provides a unique steady flame solution within one fourth and half of $l_D$, $l_D$ can be adjusted to tune $\tau _{res}$. The same approach has been applied to study 1D auto-igniting flames in Schulz and Noiray (2019a). For sufficiently high inlet temperature, conventional deflagration waves turn into auto-ignition waves when $\tau _{res}$ approaches $\tau _{AI}$. Figure 5 shows the values of $\alpha$, $\phi _{\omega }$ and $\phi _{s}$ for several values of the $\frac{\tau _{res}}{\tau _{AI}}$ ratio. The combustion mode indicator $\alpha$ is able to correctly retrieve the change of combustion regime: $|\alpha | \le 1$ regions appear upstream of the flame in pure auto-ignition regimes, while zones where diffusion promotes ignition ($\alpha > 1$) appear upstream of the flame in deflagration regimes. In between, mixed propagation modes with intermediate values of $\alpha$ are also possible, for which the auto-ignition chemistry supports flame propagation.

## Numerical Results

### Combustion Regimes

**Fig. 6:**
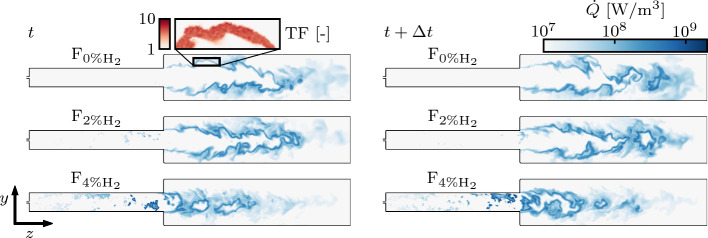
Planar cuts colored by the heat release rate ${\dot{Q}}$. From top to bottom: cases F_0%H2_, F_2%H2_, and F_4%H2_. Two different instants separated by $\Delta t=0.5$ ms are represented per case, after a statistically steady state has been reached. The inset shows the Thickening Factor (TF) field

The heat release rate (${\dot{Q}}$) field on a central plane of the domain is shown in Fig. 6 for F_0%H2_ , F_2%H2_, and F_4%H2_. The use of a 4% ${\text{H}}_{2}$ blend (F_4%H2_) has clear effects on the chemical activity in the SB and on the shape of the flame in the CC. Multiple auto-ignition kernels are continuously initiated in the SB and advected downstream where they merge with the flame front in the CC. This effect becomes less evident as the amount of ${\text{H}}_{2}$ in the fuel blend drops: weak chemical activity is detected by the heat release rate field in the SB for the F_2%H2_ case, while no activity is observed for F_0%H2_, where combustion only occurs in the CC. The power released, on average, by means of auto-igniting kernels in the SB can be quantified evaluating the $\overline{{\mathcal {P}}}_{\%}(z)$ metric (Eq. (9)) at the outlet of the SB. In particular, approximately 23% of the power is generated via auto-ignition kernels in the SB for F_4%H2_, 5% for F_2%H2_, and 0% for F_0%H2_. From these observations, it can be presumed that in F_0%H2_ the flame combustion regime is dominated by propagation via the back-diffusion of heat and radicals, while auto-ignition chemistry progressively plays a more important role with a strong effect on the stabilization of the flame in the CC as the ${\text{H}}_{2}$ content is increased.

Analysis of the CEM is now performed to clearly identify the combustion regimes involved in the flame stabilization. While the application of CEMA (Sect. 2.3) on a Direct Numerical Simulation (DNS) is straightforward, the computation of the Jacobian matrix $\mathbf {J_\omega }$ (Eq. (2)) in a LES context relies on filtered quantities that can introduce biases into the analysis. Subgrid turbulence-chemistry interaction is neglected in the context of CEMA, namely it is assumed that $\widetilde{\varvec{\omega }}=\varvec{\omega }(\widetilde{\varvec{y}})$, where ${\widetilde{\cdot }}$ is used to indicate the filtered quantities. Conversely, sub-grid scale effects are included when computing the non-chemical source term ${\varvec{s}}$ (Eq. (7)), taking into account the contributions from both molecular and turbulent diffusion. CEMA has already been applied to data from LES (Schulz et al. 2019b; Malé et al. 2022). Recently, a systematic discussion of the extension of the CEMA framework to LES has been proposed in (Liu et al. 2023). In the present study, a sensitivity study of the results to the mesh cell size has been performed to ensure the validity of the approach, and is reported as Supplementary Material. The results of the analysis are not impacted by a finer grid resolution.

Before employing CEMA to scrutinize the system properties, it is crucial to discuss some of the limitations of this approach. Firstly, CEMA specifically focuses on chemical processes, with the CEM being purely a chemical property of the system. Non-chemical processes may still play a role in system’s evolution, consequently making the existence of a CEM not always result in mixture ignition. Given that the CEM remains a necessary condition for ignition, however, CEMA still provides insights into the explosive tendency of the mixture. Secondly, CEMA implicitly assumes the relevance of CEM compared to the other modes. This assumption may not necessarily hold in a non-linear context. The validity of this assumption is addressed in Appendix 1 using a 0D well-mixed adiabatic configuration. It is important to note, however, that this approach proves the relevance of the CEM exclusively on the chemical standpoint, due to the absence of transport and mixing phenomena that characterize the 3D LES.

**Fig. 7:**
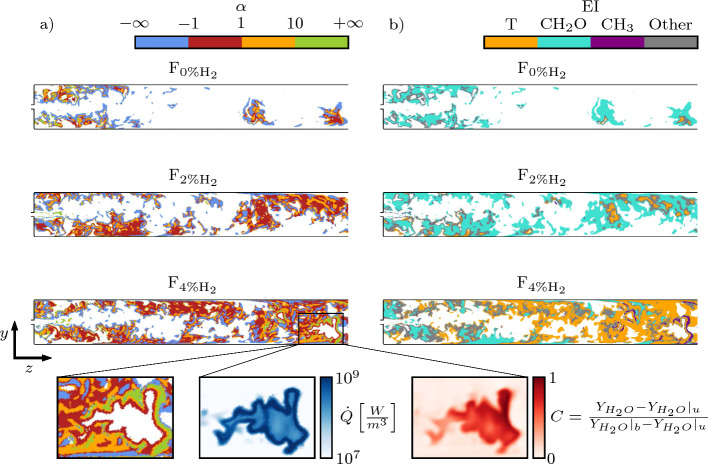
Planar cuts colored by ${\alpha }$ (Eq. (8)) (**a**) and ${\varvec{{\text{EI}}}}$ (Eq. (4)) largest entry (**b**), for F_0%H2_ (top) , F_2%H2_ (middle), and F_4%H2_ (bottom). The zoomed insets highlight the details of $\alpha$, the heat release rate ${\dot{Q}}$, and a progress variable C around an auto-ignition kernel. The progress variable C depicts the presence of a burned gas pocket. ${Y_{H_{2O}}}|_{u}$ and ${Y_{H_{2O}}}|_{b}$ represent the unburned and burned H_2_O mass fractions, respectively, assuming combustion occurs at perfect mixing conditions

The combustion mode indicator ${\alpha }$ and the largest ${\textbf{EI}}$ entry associated locally to the CEM are shown in Fig. 7 on a central plane of the domain for explosive mixtures. The advected mixture in the SB has limited time to be driven away from equilibrium before it is eventually burned at the main flame brush location in the combustion chamber, becoming non-explosive. Therefore, a minimum threshold value of $\lambda ^{thr} = 100~{\mathrm {s^{-1}}}$ is set for $\Re \left( \lambda _e\right)$ to take into consideration only the CEMs whose characteristic times $1 / \Re \left( \lambda _e\right)$ are lower or comparable to the sequential burner residence time. Only a single instant is represented in Fig. 7 since the variations in the ${\alpha }$ and ${\textbf{EI}}$ fields are only due to the unsteady nature of the turbulent flow, making the conclusions independent of the specific snapshot considered.

Unexpected explosive tendency of the mixture is observed near the fuel injector body in all the cases, as revealed by values of $\Re \left( \lambda _e\right)$ above $\lambda ^{thr}$. In the F_0%H2_ case, this explosive tendency disappears further downstream in the SB. The absence of fast enough CEM upstream of the main flame brush highlights a limited influence of auto-ignition chemistry in supporting flame propagation in the CC. The flame anchors at the exit of the SB thanks to the recirculation zones Z2 depicted in Fig. 2. In the F_2%H2_ case, fast CEMs (i.e., $\Re \left( \lambda _e\right) \ge \lambda ^{thr}$) are observed in larger regions of the SB. The ${\alpha }$ field shows that chemistry starts to play a role in the ignition process governed by the CEM (${|\alpha |<1}$). The largest ${\textbf{EI}}$ entry for F_0%H2_ and F_2%H2_ is mainly represented by formaldehyde CH_2_O. CH_2_O is a precursor of methane auto-ignition and is typically produced in relatively large amount before ignition occurs (Gordon et al. 2009; Li and Williams 2002). The dominance of CH_2_O in the ${\textbf{EI}}$ shows the tendency of the chemical kinetics to oxidize ${\text{CH}}_{4}$ via radicals where the fuel and the hot vitiated air stream first mix and in the SB. However, chemistry is not fast enough to continuously trigger ignition kernels. F_4%H2_ features fast CEMs (i.e., $\Re \left( \lambda _e\right) \ge \lambda ^{thr}$) in almost the entire SB. Analysis of the largest ${\textbf{EI}}$ entry indicates a reactive mixture that transitions to thermal runaway before eventually igniting within the SB, generating kernels of burned gases. The ${\alpha }$ field highlights the dominant role of chemistry in the ignition process. While the kernels are advected towards the end of the SB, diffusion starts to play a dominant role close to the kernel boundaries, as shown in the zoomed inset in Fig. 7. This demonstrates that the kernel fronts develop to propagating flames as they grow in the SB, before merging with the main flame brush. A similar phenomenon has already been observed during plasma-induced ignition in a sequential burner (Malé et al. 2022). Here, earlier ignition in the SB is caused by the ${\text{H}}_{2}$ addition. This detailed ignition sequence confirms that auto-ignition chemistry plays an important role upstream of the main flame brush for F_4%H2_, contributing to the stabilization of the flame in the sequential combustion chamber.

### Fuel Oxidation Mechanisms

**Fig. 8:**
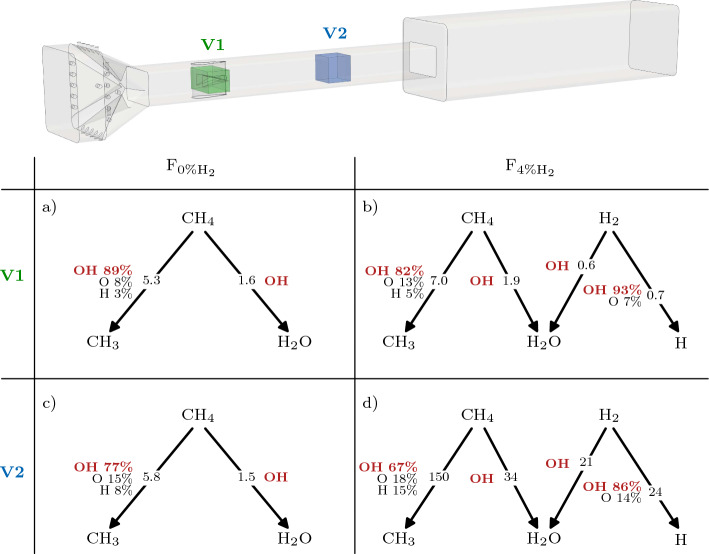
RPA diagrams following the H atom for ${\text{F}}_{{0\%{{\rm H}}_{2}}}$ (**a**, **c**) and ${\text{F}}_{{4\%{{\rm H}}_{2}}}$ (**b**, **d**). Diagrams on the top (**a**, **b**) represent location V1; diagrams on the bottom (**c**, **d**) represent location V2. Fluxes units are kmol/${\mathrm {m^3}}$/s. Only the fluxes involved in the direct decomposition of the fuel molecules are represented

Reaction Path Analysis (RPA) is used to investigate the mechanisms leading to fuel decomposition in the SB. The discussion focuses on the two extreme cases F_0%H2_ and F_4%H2_ for brevity, given that the F_2%H2_ case provides intermediate results. The objective of this analysis is to identify the chemical pathways leading to fuel decomposition when: i) the fuel comes into contact with the hot vitiated air and ii) later in the SB. Two volumes are defined for this purpose: one in the recirculation zone attached to vortex generators of the fuel injector (V1); and the other in the middle of the SB (V2). Reaction rates are averaged over time and space in the volumes V1 and V2 as

11 $$\begin{aligned} \overline{{\mathcal {Q}}_j} = \frac{1}{t^{av} V} \int _0^{t^{av}} \iiint _{V} {\mathcal {Q}}_j (x,y,z,t) \, dx \, dy \, dz \, dt \, \text {,} \end{aligned}$$

where ${\mathcal {Q}}_j$ is the rate of progress of reaction *j*, V is the averaging volume (V1 or V2) and $t^{av} = 10$ ms is the averaging time. Figure 8 shows the results of the RPA, with a visual representation of the volumes V1 and V2. Only the paths responsible for the direct decomposition of the fuel molecules are shown. The decomposition of ${\text{CH}}_{4}$ to CH_3_ is the most important flux for all the cases/volume locations.

The RPA diagrams at V1 are similar between F_0%H2_ and F_4%H2_ in terms of the species involved in fuel decomposition, and of the magnitude of the fluxes. The decomposition of the fuel molecules to CH_3_, H and H_2_O is mainly due to the action of the OH radicals. Indeed, the reactions of fuel with OH, H or O are known to be the fastest ones responsible for fuel breakup at high temperatures, once a radical pool has been formed (Glassman et al. 2015). In the present configuration, the radical pool is provided by the first stage combustion. A significant amount of OH is found upstream of the fuel injector, where its average mass fraction is of about ${10^{-4}}$.

The diagrams at V2 show that, while the OH radicals are still highly involved in the decomposition of the fuel molecules, the atomic H and O play a more important role compared to V1. This is mainly due to the chemical activity near the fuel injector that partially consumes the OH reservoir advected from the first stage and promotes the formation of other radicals and dissociated fuel products. Significant differences appear in terms of magnitude of the fluxes between ${\text{F}}_{{0\%{{\rm H}}_{2}}}$ and ${\text{F}}_{{4\%{{\rm H}}_{2}}}$. The fluxes have the same order of magnitude in V1 and V2 for ${\text{F}}_{{0\%{{\rm H}}_{2}}}$, while they are more than an order of magnitude larger in V2 than in V1 for ${\text{F}}_{{4\%{{\rm H}}_{2}}}$. This quantifies the higher chemical activity of the ${\text{CH}}_{4}$-${\text{H}}_{2}$ mixture in the SB, that will eventually lead to auto-ignition.

### Vitiated Flow’s Relaxation Towards Equilibrium

In the first stage of a sequential combustor, OH radicals are produced and flow towards the dilution air mixer. The OH mass fraction found at the second stage fuel injector location, however, may significantly differ from the one just upstream of the dilution air mixer due to the interaction with the cold dilution air jets. The injection of cold air modifies both enthalpy and mixture composition, shifting the chemical equilibrium point of the mixture. Chemical activity is therefore playing an important role in the vitiated flow as it evolves towards the new chemical equilibrium point upstream of the sequential fuel injector. The interplay between the chemical kinetics and the residence time between the two stages determines whether this chemical relaxation process can be completed before the vitiated air reaches the second stage fuel injection location.

**Fig. 9:**
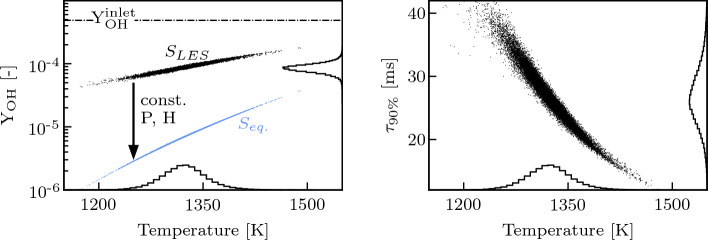
Left: Scatter plot representing in the OH-temperature subspace the thermochemical states extracted from the LES on a plane right upstream of the fuel injector ($S_{LES}$). The point distribution is projected on both axes. The corresponding equilibrium states ($S_{eq.}$) (in blue) are obtained at constant enthalpy and pressure from $S_{LES}$. Right: Scatter plot representing the time $\tau _{90\%}$ needed to cover 90% of the distance between the sampled and the equilibrium OH mass fractions

Given the importance of OH on the ignition chemistry, its mass fraction ${\text{Y}}_{{\textrm{OH}}}$ is taken as reference to quantify the distance of the advected mixture from chemical equilibrium. Individual thermochemical states ($S_{LES}$) are extracted on a plane right upstream of the fuel injector from multiple instantaneous LES solutions and plotted in the OH-temperature subspace in Fig. 9, where they are compared with:

- the frozen chemistry line ${\text{Y}}_{{\textrm{OH}}}^{{\textrm{inlet}}}$, which represents the mass fraction of OH injected at the inlet of the domain, with the assumption that the residence time in the first stage chamber (not simulated in this work) is long enough for having reached chemical equilibrium;
- the equilibrium states $S_{eq.}$ computed assuming constant pressure and enthalpy from the thermochemical states sampled from the LES, which represent the conditions reached in case of fast enough chemistry once the inlet flow and the dilution air have perfectly mixed.

The LES data highlight that the equilibrium is not reached: there is still margin for the OH mass fraction to decrease (Fig. 9). ${\text{Y}}_{{\textrm{OH}}}$ can span more than two orders of magnitude between its value at the inlet and at equilibrium, suggesting that the combustion behavior of the sequential combustor can be strongly affected by the distance of the vitiated air from chemical equilibrium, as will be confirmed in Sect. 3.4. Parameters influencing the relaxation towards equilibrium just upstream of the sequential fuel injection become therefore crucial for the operability of this type of combustors, including not only the thermodynamic properties of the system (i.e., the operating point), but also the combustor geometry, which directly influences the residence time and dilution air mixing process between the two stages. As depicted in Fig. 9, the time needed to cover 90% of the distance between the sampled and the equilibrium OH mass fraction does not exceed 10 times the residence time in the combustor. Therefore, investigating the effects of the vitiated flow’s relaxation towards chemical equilibrium is physically relevant for this type of combustor architecture.

### Influence of Equilibrium on the Ignition Process

The effects of chemical equilibrium on the ignition behavior of the sequential stage are investigated via an additional ad-hoc LES labelled ${\text{F}}_{{4\%{{\rm H}}_{2}}^*}$. The ${\text{F}}_{{4\%{{\rm H}}_{2}}^*}$ conditions are artificially tuned to represent the limit case where the air-diluted vitiated flow has enough time to attain equilibrium composition before reaching the second stage fuel injector. This is achieved according to the following methodology:

1. the average thermochemical state of the diluted, vitiated flow right upstream of the fuel injector is extracted from the LES, corresponding to the average state of the $S_{LES}$ point cloud in Fig. 9;
2. from these conditions, the corresponding equilibrium state at constant enthalpy and pressure is computed;
3. the composition imposed at the inlet in the LES is adjusted to retrieve, on average, this equilibrium state, taking into account only mixing with the dilution air and assuming no chemical reaction takes place;
4. given that chemical reactions are not taken into account when tuning the inlet composition, chemistry is artificially deactivated in the LES between the main inlet and the fuel injector.

This approach results in a stratified mixture upstream of the fuel injector that features, on average, an amount of OH radicals comparable to the equilibrium distribution $S_{eq.}$ in Fig. 9. ${\text{F}}_{{4\%{{\rm H}}_{2}}}$ and ${\text{F}}_{{4\%{{\rm H}}_{2}}^*}$ are compared in terms of chemical activity and CEMA quantities in Secs 3.4.1 and 3.4.2.

#### Chemical Activity in the Sequential Burner

**Fig. 10:**
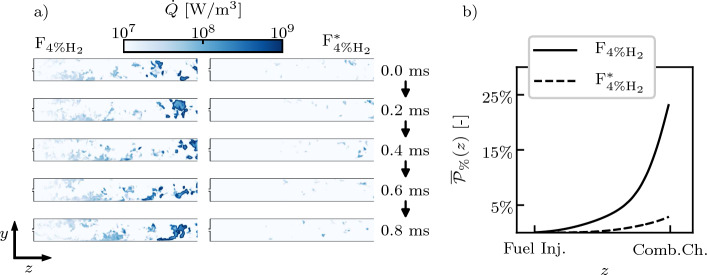
Planar cuts of the sequential burner colored by the heat release rate ${\dot{Q}}$ for ${\text{F}}_{{4\%{{\rm H}}_{2}}}$ (left column) and ${\text{F}}_{{4\%{{\rm H}}_{2}}^*}$ (right column) for multiple instants spaced by 0.2 ms (**a**) and profiles of $\overline{{\mathcal {P}}}_{\%}(z)$ (Eq. (9)) in the sequential burner as function of the streamwise coordinate (**b**)

Fig. 10a highlights significant differences between ${\text{F}}_{{4\%{{\rm H}}_{2}}}$ and ${\text{F}}_{{4\%{{\rm H}}_{2}}^*}$ in terms of the generation of auto-ignition kernels in the SB. While ${\text{F}}_{{4\%{{\rm H}}_{2}}}$ features an intense production of auto-ignition kernels in multiple regions of the SB, ${\text{F}}_{{4\%{{\rm H}}_{2}}^*}$ is characterized by much weaker auto-ignition kernel formation, limited to some sporadic events towards the end of the SB. Fig. 10b represents the time-averaged normalized total power released up to *z*, $\overline{{\mathcal {P}}}_{\%}(z)$ (Eq. (9)). The trends depicted in the 2D fields are confirmed on a longer timescale and on the whole 3D domain, highlighting that the release of heat by combustion occurs right after the fuel injector in ${\text{F}}_{{4\%{{\rm H}}_{2}}}$, while it is postponed towards the end of the SB for ${\text{F}}_{{4\%{{\rm H}}_{2}}^*}$. The final value reached by $\overline{{\mathcal {P}}}_{\%}(z)$ at the end of the SB quantifies the power released, on average, in the SB: approximately 23 % of the total fuel power is released by means of auto-igniting kernels in ${\text{F}}_{{4\%{{\rm H}}_{2}}}$, while this percentage drops to approximately 3 % in ${\text{F}}_{{4\%{{\rm H}}_{2}}^*}$.

**Fig. 11:**
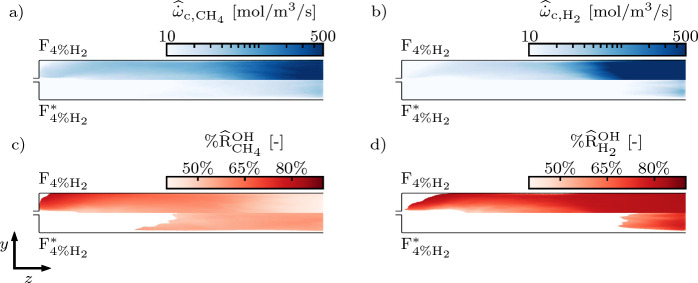
Planar cuts of the SB colored by the ${\text{CH}}_{4}$ consumption rate $\widehat{\dot{\omega }}_{\textrm{c,CH}_{\textrm{4}}}$ (**a**), the ${\text{H}}_{2}$ consumption rate $\widehat{\dot{\omega }}_{\textrm{c,H}_{\textrm{2}}}$ (**b**), ${\%\widehat{{\text{R}}}_{{\text{CH}}_{4}{}}^{\rm OH}}$ (**c**), and ${\%\widehat{{\text{R}}}_{{\text{H}}_{2}{}}^{\rm OH}}$ (**d**). ${\%\widehat{{\text{R}}}_{{\text{CH}}_{4}{}}^{\rm OH}}$ and ${\%\widehat{{\text{R}}}_{{\text{H}}_{2}{}}^{\rm OH}}$ are represented only in the regions where $\widehat{\dot{\omega }}_{\textrm{c,CH}_{\textrm{4}}}$ and $\widehat{\dot{\omega }}_{\textrm{c,H}_{\textrm{2}}}$ are above the threshold value of 10 mol/m^3^/s. The fields are the result of a 10 ms average

Figure 11 depicts the time-averaged consumption rates of ${\text{CH}}_{4}$ $\widehat{\dot{\omega }}_{\textrm{c,CH}_{{4}}}$ and ${\text{H}}_{2}$ $\widehat{\dot{\omega }}_{\textrm{c,H}_{{2}}}$ in the SB. They are computed summing the contributions of all the reactions characterized by negative stoichiometric coefficients for the fuel molecules. Reversible reactions are split into forward and backward reactions that contribute individually to the sums. ${\%\widehat{{\text{R}}}_{{\text{CH}}_{4}{}}^{{\rm OH}}}$ and ${\%\widehat{{\text{R}}}_{{\text{H}}_{2}{}}^{{\rm OH}}}$ quantify the relative contributions of reactions ${{\text{R}}_{{\text{CH}}_{4}{}}^{{\rm OH}}}$ (${{\text{CH}}_4 + {\textrm{OH}} \to {\text{CH}}_3 + {\text{H}}_{2}{\textrm{O}}}$) and ${{\text{R}}_{{\text{H}}_{2}{}}^{{\rm OH}}}$ (${{\text{H}}_2 + {\textrm{OH}} \to {\textrm{H}} + {\text{H}}_\textrm{2}{\textrm{O}}}$) to $\widehat{\dot{\omega }}_{\textrm{c,CH}_{\textrm{4}}}$ and $\widehat{\dot{\omega }}_{\textrm{c,H}_{\textrm{2}}}$, respectively. The averaging operation is performed over 10 ms.

In ${\text{F}}_{{4\%{{\rm H}}_{2}}}$, the out-of-equilibrium flow composition promotes the formation of a chemically active region in the recirculation zone attached to the fuel injector (Z1 in Fig. 2) where both ${\text{CH}}_{4}$ and ${\text{H}}_{2}$ molecules are decomposed. In agreement with the analysis in Sect. 3.2, OH is the main responsible for fuel consumption with the reactions ${{\text{R}}_{{\text{CH}}_{4}{}}^{{\rm OH}}}$ and ${{\text{R}}_{{\text{H}}_{2}{}}^{{\rm OH}}}$ accounting for more than 80% of $\widehat{\dot{\omega }}_{\textrm{c,CH}_{\textrm{4}}}$ and $\widehat{\dot{\omega }}_{\textrm{c,H}_{\textrm{2}}}$, respectively. Proceeding downstream in the SB, part of the excess OH advected from the first stage is consumed and a pool of active radicals and intermediate species is progressively built: new pathways of ${\text{CH}}_{4}$ decomposition are available and ${\%\widehat{{\text{R}}}_{{\text{CH}}_{4}{}}^{\rm OH}}$ decreases to roughly 50%. The ${\text{H}}_{2}$ consumption pathways are less affected by this behavior and ${\%\widehat{{\text{R}}}_{{\text{H}}_{2}{}}^{\rm OH}}$ remains above 70% proceeding towards the end of the SB.

Conversely, in ${\text{F}}_{{4\%{{\rm H}}_{2}}^*}$, weaker fuel decomposition is taking place close to the fuel injector compared to ${\text{F}}_{{4\%{{\rm H}}_{2}}}$, with a slight increase towards the end of the SB. Both $\widehat{\dot{\omega }}_{\textrm{c,CH}_{\textrm{4}}}$ and $\widehat{\dot{\omega }}_{\textrm{c,H}_{\textrm{2}}}$ are approximately one order of magnitude smaller than in ${\text{F}}_{{4\%{{\rm H}}_{2}}}$. Furthermore, the relative importance of OH is reduced, especially for CH_4_ decomposition where ${\%\widehat{{\text{R}}}_{{\text{CH}}_{4}{}}^{\rm OH}}$ is approximately 60%.

#### Analysis of the Explosive Modes

The ${\text{F}}_{{4\%{{\rm H}}_{2}}}$ and ${\text{F}}_{{4\%{{\rm H}}_{2}}^*}$ results are now analyzed with respect to their CEM. 0D reactor simulations computed with Cantera (Goodwin et al. 2022) are used to support the analysis. The initial state of the 0D simulations is set by mixing fuel and vitiated air. The vitiated air composition is varied to construct three scenarios: ${\text{R}}_{{4\%{{\rm H}}_{2}}}$, ${\text{R}}_{{4\%{{\rm H}}_{2}}^{*}}$ and ${\text{R}}_{{4\%{{\rm H}}_{2}}^{0}}$. ${\text{R}}_{{4\%{{\rm H}}_{2}}}$ and ${\text{R}}_{{4\%{{\rm H}}_{2}}^{*}}$ mimic the ${\text{F}}_{{4\%{{\rm H}}_{2}}}$ and ${\text{F}}_{{4\%{{\rm H}}_{2}}^*}$ cases, respectively. Therefore, the thermochemical state of the vitiated air is set at the average conditions right upstream of the fuel injector for ${\text{R}}_{{4\%{{\rm H}}_{2}}}$, while it is set at chemical equilibrium for ${\text{R}}_{{4\%{{\rm H}}_{2}}^{*}}$. ${\text{R}}_{{4\%{{\rm H}}_{2}}^{0}}$ represents a limit case where the vitiated flow consists only of major species (i.e., N_2_, O_2_, H_2_O and CO_2_). The 0D reactors are adiabatic and isobaric.

**Fig. 12:**
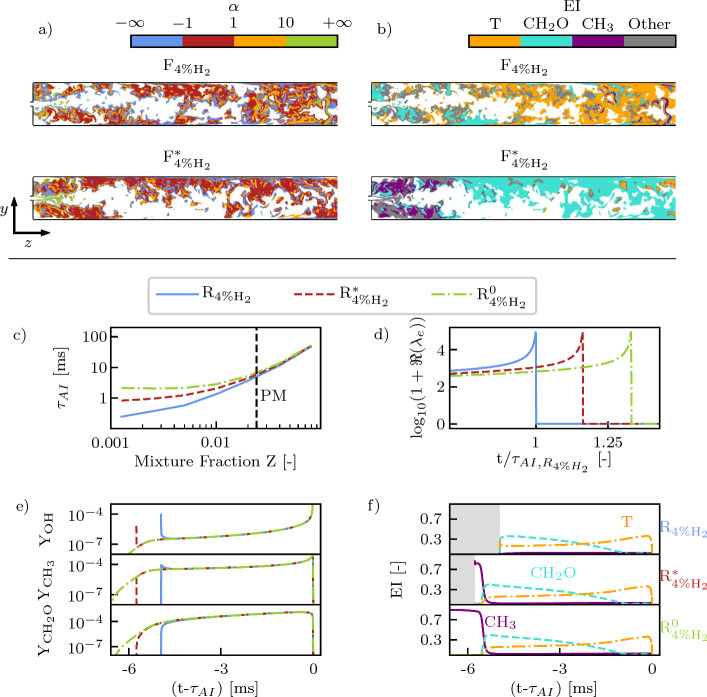
Planar cuts colored by ${\alpha }$ (Eq. (8)) (**a**) and **EI** (Eq. (4)) largest entry (**b**) for F_4%H2_ (top) and F_4%H2∗_ (bottom). Results of CEMA for 0D reactors in terms of: auto-ignition delay $\tau _{AI}$ as function of the mixture fraction between fuel and vitiated air (**c**); real part of CEM eigenvalue $\Re (\lambda _e)$ (Eq. (3)) (**d**); profiles of OH, CH_3_ and CH_2_O mass fractions as function of time (**e**); EI of temperature, CH_2_O and CH_3_ as function of time (**f**). The time coordinate in (**e**) and (**f**) is shifted by $\tau _{AI}$ to make all reactors ignite at 0 ms. The vertical, dashed line in (**c**) indicates the Perfectly Mixed (PM) mixture fraction

The results of the 0D reactor analysis are depicted in Figs 12c-f. Figures 12d-f are generated assuming perfect mixing between the fuel and the vitiated air. As highlighted in Fig. 12e, the presence of radicals in the vitiated air influences only the beginning of the ignition processes by quickening the initial fuel-decomposition and radical build-up phase. After the initial transient, no difference emerges in the evolutions of the states of ${\text{R}}_{{4\%{{\rm H}}_{2}}}$, ${\text{R}}_{{4\%{{\rm H}}_{2}}^{*}}$ and ${\text{R}}_{{4\%{{\rm H}}_{2}}^{0}}$. The initial fuel decomposition phase features CH_3_ as the dominating species of the CEM (Fig. 12f). The CH_3_-dominated phase appears in both the ${\text{R}}_{{4\%{{\rm H}}_{2}}^{*}}$ and R_4%H20_ reactors, while it is completely bypassed in R_4%H2_ due to the high presence of radicals in the vitiated air. The additional time spent by the R_4%H2∗_ and R_4%H20_ mixtures in the CH_3_-dominated phase leads to an increase in the ignition delay compared to R_4%H2_. The same trend is observed also for other mixture fractions around the perfectly mixed one (Fig. 12c). In particular, the leaner the mixture, the larger the relative effect of the vitiated air composition on the ignition delay.

These results can be linked with the ones observed in F_4%H2_ and F_4%H2∗_. The fields of $\alpha$ and of the largest $\textbf{EI}$ entry are represented in the planar cuts in Figs 12a and b only in the regions where $\Re \left( \lambda _e\right) \ge \lambda ^{thr}$, indirectly highlighting that $\Re \left( \lambda _e\right)$ is above the threshold value in large portions of the domain also in F_4%H2∗_. This is expected also from the 0D analysis (Fig. 12d), where the vitiated flow composition has almost no influence on the $\Re \left( \lambda _e\right)$ value before ignition. The extended $|\alpha |< 1$ regions upstream of the flame front highlight that the explosive mode is dominated by the chemical source term almost everywhere in F_4%H2∗_. The auto-ignition chemistry is therefore playing a role in the unburned gases. Nevertheless, the increase in ignition delay highlighted in Fig. 12c hinders the capability of the advected mixture to form auto-ignition kernels within the physical boundaries of the SB. The advected mixture has not enough time to react upstream of the main flame brush, leading to a weaker impact of auto-ignition chemistry on the main flame stabilization mechanism in F_4%H2∗_ as opposed to F_4%H2_. The $\mathrm {\textbf{EI}}$ field suggests that the chemical activity in F_4%H2∗_ is mainly associated with the radical build-up phase by means of fuel oxidation. In analogy with R_4%H2∗_, CH_3_-dominated regions appear close to the fuel injector in F_4%H2∗_, followed by CH_2_O-dominated regions that extend in the whole SB. As opposed to the F_4%H2_ case and in agreement with the auto-igniting kernel suppression already discussed, a transition to the thermal runaway phase is not observed within the SB in F_4%H2∗_.

## Conclusions

The present paper reports the results of a numerical investigation on the ignition and combustion behavior of pure ${\text{CH}}_{4}$ fuel (case F_0%H2_) and of two ${\text{H}}_{2}$-enriched ${\text{CH}}_{4}$ fuel blends (cases F_2%H2_ and F_4%H2_) in the second stage of a sequential combustor, performed using LES in combination with a precise description of the chemistry. It is found that, for the operating conditions considered, replacing 4% in mass of ${\text{CH}}_{4}$ with ${\text{H}}_{2}$ (F_4%H2_) significantly changes the behavior of the sequential flame, causing auto-ignition events in the sequential burner, upstream of the sequential flame. This effect is weaker if only 2% of fuel mass is replaced by ${\text{H}}_{2}$ (F_2%H2_): approximately 5 times less fuel burns in auto-ignition mode upstream of the combustion chamber compared to F_4%H2_. Analysis of the explosive modes highlights increased chemical activity and explosiveness of the mixture upstream of the main flame brush as the amount of ${\text{H}}_{2}$ is increased. Regions undergoing thermal runaway are clearly identified in the F_4%H2_ case. The combustion modes in the sequential burner and chamber are strongly influenced by minor combustion products advected from the first stage, especially OH radicals. Indeed, as shown via Reaction Path Analysis (RPA), they induce a prompt fuel oxidation near the fuel injector, enhancing the chemical activity in the sequential burner and ultimately leading to the formation of auto-ignition kernels in the F_4%H2_ case. This is primarily due to the large concentration of OH upstream of the fuel injector, whose mass fraction is, on average, approximately an order of magnitude higher than that of the theoretical chemical equilibrium reached by assuming a perfect mixing between the vitiated and the dilution air. The importance of the out-of-equilibrium composition in determining the combustion mode of the second stage is demonstrated performing an additional ad-hoc LES (F_4%H2∗_) characterized by the vitiated air flow at chemical equilibrium upstream of the fuel injector. Compared to F_4%H2_, F_4%H2∗_ features weaker fuel oxidation in the sequential burner and the inhibition of auto-ignition kernels formation. Therefore, the parameters affecting the relaxation towards chemical equilibrium of the vitiated air flow are expected to interfere with the behavior of sequential combustors operating at auto-ignition conditions with varying fractions of ${\text{H}}_{2}$ blending. These include both the thermodynamic conditions, which define the equilibrium point, and the combustor geometry, which affects the residence time and determines whether the equilibrium can be reached. The findings of this study will be useful for the development of sequential combustors capable of switching from pure natural gas to pure hydrogen without compromising on the very low pollutant emissions and on the high combustor outlet temperature targets. They highlight the combustion regime sensitivity to both fuel composition and vitiated air relaxation towards chemical equilibrium, which can have consequences on pollutant formation, acoustics, and mechanical integrity of the system.

### Supplementary Information

Below is the link to the electronic supplementary material.

Supplementary file 1 (pdf 480 KB)

## Figures and Tables

**Fig. 13:**
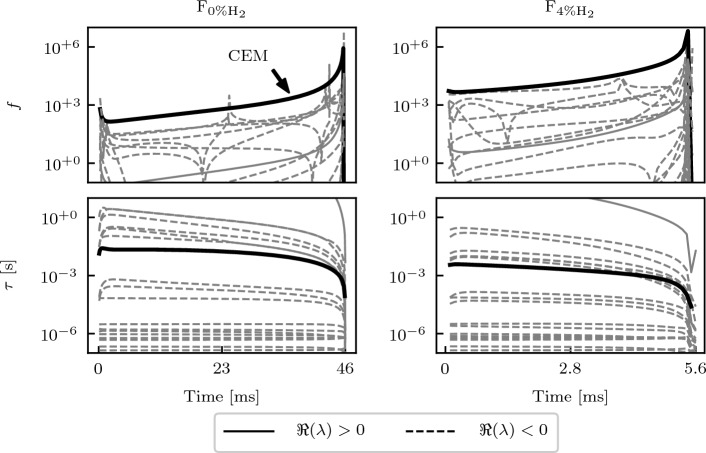
Amplitudes *f* (top) and time-scales $\tau$ (bottom) of the chemical modes in 0D reactors initialized at perfect mixing thermochemical conditions representative of F_0%H2_ (left) and F_4%H2_ (right). A thick, black line is used to highlight the CEM

## Data Availability

Data can be made available upon request to the corresponding author.

## Appendix 1: Relevance of the Chemical Explosive Mode

In zero-dimensional (0D) systems, the Chemical Explosive Mode (CEM) analyzed using Chemical Explosive Mode Analysis (CEMA) is by definition one of the Computational Singular Perturbation (CSP) slow modes. While in principle all the slow modes should be accounted for when studying the evolution of a reactive mixture to capture its multi-scale nature, this feature is overlooked within the CEMA framework where only the CEM (i.e., the fastest explosive mode) is analyzed. To justify the use of CEMA, therefore, it must be proven that the CEM plays a dominant role in determining the evolution of the system. For this reason, the interplay between the CEM and the other chemical modes is investigated using tools from CSP (Lam and Goussis 1989) and the PyCSP python package (Malpica Galassi 2022). The analysis is performed in 0D Cantera (Goodwin et al. 2022) reactors where the initial state is defined assuming perfect mixing between the fuel and the vitiated air at the average thermochemical conditions right upstream of the fuel injector in the LES. The fuel composition is representative of F_0%H2_ and F_4%H2_. Similar results are obtained for F_2%H2_, omitted here brevity.

The relevance of the CEM can be implied if: it has the largest amplitude *f* among all the modes and the system dynamics is quasi-linear (Tingas et al. 2017). Figure 13 clearly highlights that the amplitude requirement is met in the present configuration. The quasi-linear dynamics of the system is suggested by the approximate constancy of the time-scales of all the modes except the slowest ones. Following a similar approach as in Tingas et al. (2017), the quasi-linear nature of the system can be further verified analyzing the sensitivity of the ignition delay time of the mixtures to the rates of the reactions featuring the largest Timescale Participation Index (TPI) relative to the CEM. Two reactions are chosen for this purpose: R12 (${{\textrm{O}}_\textrm{2} + {\textrm{H}} \to {\textrm{O}} + {\textrm{OH}}}$) and R72 (${{\text{CH}}_{4} + {\textrm{H}} \to {\text{CH}}_{3} + {\text{H}}_{2}}$). R12 is a chain branching reaction and features the largest positive TPI (Goussis and Najm 2006) associated to the CEM during the thermal runaway phase at both F_0%H2_ and F_4%H2_ conditions. R72, on the contrary, is a chain termination reaction that features the smallest negative TPI during the aforementioned thermal runaway phase. The analysis is performed perturbing the rates of these reactions by 20% and 40% (Table 2). The amplitude of the ignition delay variation $\Delta \tau _{AI}$ scales almost linearly with the amplitude of the imposed perturbations. Furthermore, $\Delta \tau _{AI}$ has always the opposite sign compared to the TPI, highlighting that the qualitative response of the system agrees with the explosive (${\textrm{TPI}}>0$) or dissipative (${\textrm{TPI}}<0$) influence of each reaction on the CEM.

**Table 2:** Results of the reaction rate constant perturbation analysis

Reaction	TPI	Perturb	${{\textbf {F}}_{{0\%\textbf{H}}_{2}}}$	${{\textbf {F}}_{{4\%\textbf{H}}_{2}}}$
			$\Delta \tau _{AI}$ (%)	$\Delta \tau _{AI}$ (%)
${{\textrm{O}}_{2} + {\textrm{H}} \rightarrow {\textrm{O}} + {\textrm{OH}}}$	$>0$	$1.2\cdot k$	$-$7.6	$-$23.2
		$1.4\cdot k$	$-$13.7	$-$39.3
${{\textrm{H}} + {\text{CH}}_{4} \rightarrow {\text{CH}}_{3} + {\text{H}}_{2}}$	$<0$	$1.2\cdot k$	+7.0	+5.4
		$1.4\cdot k$	+13.0	+10.7

This analysis demonstrates the importance of the CEM in determining the system slow dynamics, justifying the application of CEMA in the present work.

## Declarations

### Supplementary Information

Additional details on the Grid Sensitivity Analysis (GSA) on the diagnostic tools employed are provided as supplementary material.
