# Supplementary material for: Numerical Study of Ignition and Combustion of Hydrogen-Enriched Methane in a Sequential Combustor
Source: Flow Turbul Combust. 2024 Apr 5;112(4):1249–73. doi: 10.1007/s10494-024-00540-8 (PMC11026249; doi:10.1007/s10494-024-00540-8)
Supplement: Supplementary file 1 — (pdf 480 KB) [file 10494_2024_540_MOESM1_ESM.pdf]

Supplementary Material: Grid Sensitivity Analysis (GSA) on the diagnostic tools employed

Diagnostic tools such as Chemical Explosive Mode Analysis (CEMA) (Secs 2.3, 3.1, and 3.4.2) and Reaction Path Analysis (RPA) (Sec. 3.2) have been employed. These tools rely on the computation of chemical terms such as the reaction rates, the species source terms and the chemical Jacobian $\mathbf{J}_\omega$ (Eq. (4)). The computation of these quantities in the LES framework, however, relies on filtered variables. The impact of this approximation should be explicitly evaluated to avoid biases in the conclusions drawn with the diagnostic tools employed. The sensitivity of the diagnostic tools on the grid resolution is assessed via a Grid Sensitivity Analysis (GSA). First, the impact of grid size on the species mass fractions is analyzed in Sec. S1. Then, the results of the GSA in terms of the combustion mode indicator α (Eq. (10)) and the explosive index vector **EI** (Eq. (6)) are presented in Sec. S2. Finally, the results of the GSA in terms of the Reaction Path Analysis are presented in Sec. S3. Only the results from the $F_{4\%H_2}$ case are discussed, since this operating point features the strongest chemical activity in the SB.

S1 Species mass fractions

The influence of the grid size on the mixture composition is assessed with respect to fuel (CH_4), oxidizer (O_2), and product species (H_2O and CO_2). The $F_{4\%H_2}$ case is analyzed in two locations: upstream of the region where active kernels are formed, and close to the sequential burner outlet. As illustrated in Fig. S1, limited impact of the grid size is observed on the average mass fractions for Grid B and Grid C. Larger differences are observed for Grid A.

S2 Chemical Explosive Mode Analysis (CEMA)

Figure S2 highlights limited differences in the combustion mode indicator α (Eq. (10)) and largest Explosive Index **EI** (Eq. (6)) entry fields among the three grids used in the GSA (Table 1), for the $F_{4\%H_2}$ operating point. In terms of α, the CEM is mainly dominated by chemistry in the first part of the SB independently of the grid resolution. The transition to deflagration waves, indicated by diffusion-dominated CEM around the boundaries of the burning pockets, is well captured by all the meshes. In terms of

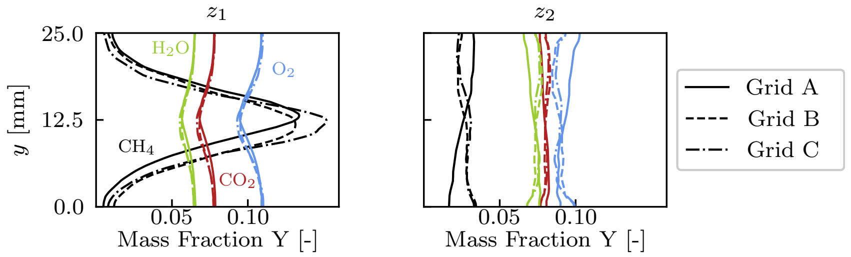

Fig. S1 Average mass fractions of CH_4 (black), H_2O (green), CO_2 (red), and O_2 (blue) along the y direction in the middle of the sequential burner, for the three different grids employed in the GSA. Location z_1 (left) is 25 mm downstream of the fuel injector. Location z_2 (right) is 25 mm upstream of the combustion chamber inlet.

the largest entry of the **EI** vector, the existence of a region dominated by CH_2O close to the fuel injector can be observed for all the grids. Additionally, the transition to the thermal runaway phase (i.e., largest **EI** entry associated to temperature) is captured by all the grids from about half of the SB. Therefore, net of the differences related to the unsteady nature of the turbulent flow, the conclusions drawn from these quantities in the manuscript are not affected by the grid size.

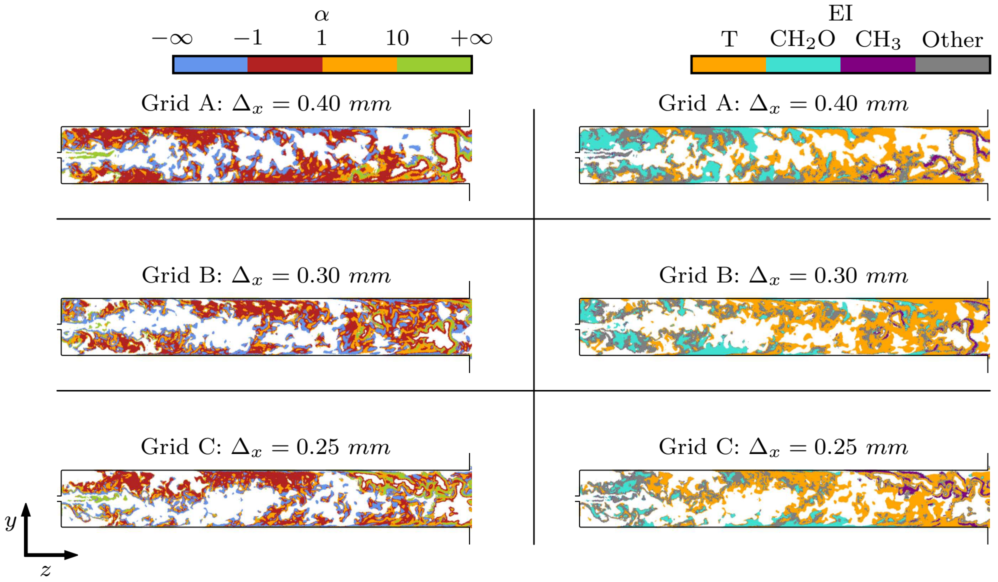

Fig. S2 Planar cuts of α (Eq. (10)) (left) and max(**EI**) variable (Eq. (6)) (right) fields in the sequential burner in the $F_{4\%H_2}$ case for the three grids used for the GSA (Table 1).

S3 Reaction Path Analysis (RPA)

Figure S3 highlights limited differences in the RPA diagrams among Grid B and Grid C (Table 1). The results for Grid A are omitted for brevity. The role of the species

in each of the fluxes is not affected by the grid resolution, although slight variations appear in the absolute values of the fluxes between the grids. For the present study, the analysis is focused on the order of magnitude of the fluxes rather than on their exact values. The conclusions drawn from these diagrams in Sec. 3.2 of the manuscript are therefore independent of the grid size.

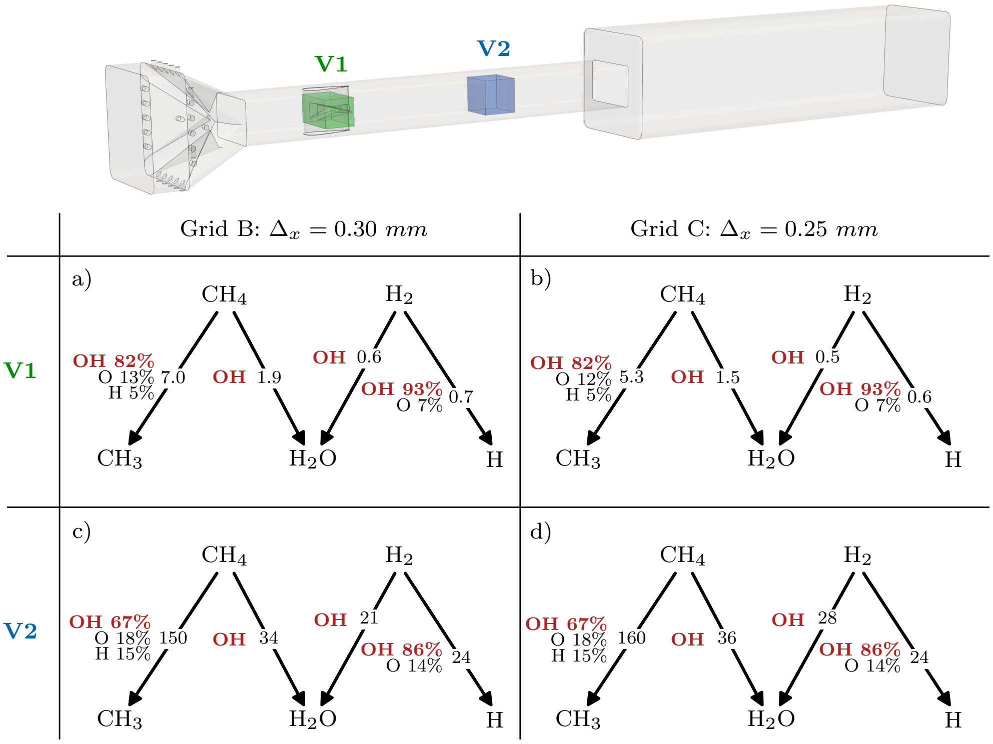

Fig. S3 RPA diagrams computed at locations V1 (a, b) and V2 (c, d) for Grid B (a, c) and Grid C (b, c) for the $F_{4\%H_2}$ operating point.
